# Weathering the storm: Do arctic blizzards cause repeatable changes in stress physiology and body condition in breeding songbirds?

**DOI:** 10.1016/j.ygcen.2018.07.004

**Published:** 2018-10-01

**Authors:** Jesse S. Krause, Jonathan H. Pérez, Helen E. Chmura, Simone L. Meddle, Kathleen E. Hunt, Laura Gough, Natalie Boelman, John C. Wingfield

**Affiliations:** a Department of Neurobiology, Physiology and Behavior, University of California Davis, One Shields Avenue, Davis, CA 95616, USA; b The Roslin Institute, The Royal (Dick) School of Veterinary Studies, The University of Edinburgh, Easter Bush, Midlothian EH25 9RG, Scotland, UK; c Northern Arizona University, Department of Biological Sciences, Flagstaff, AZ 86011, USA; d Department of Biological Sciences, Towson University, Towson, MD 21252, USA; e Department of Earth and Environmental Sciences, and Lamont-Doherty Earth Observatory of Columbia University, Palisades, NY 10964, USA

**Keywords:** Corticosterone, Hypothalamic-pituitary adrenal (HPA) axis, Climate change, Extreme events, Emergency life history stage

## Abstract

Severe weather events are increasing worldwide because of climate change. To cope with severe weather events, vertebrates rely on the stress response which is activated by the hypothalamic-pituitary adrenal (HPA) axis to adjust physiology and behavior. Previous studies have detailed changes in baseline concentrations of the stress hormone corticosterone during a single storm event, but little data exists on how stress physiology and body condition are adjusted as the storm progresses across multiple days. This represents a serious gap in our understanding of how birds respond physiologically over the duration of a storm. We documented arctic snowstorms that occurred over five consecutive years that were endured by Lapland longspurs (*Calcarius lapponicus*; 2012–2016) and in three consecutive years by white-crowned sparrows (*Zonotrichia leucophrys gambelii*; 2014–2016). Data were collected on storm-free days, during snowstorms ranging in length from 1 to 3 days, and the day immediately following a snowstorm. The specific aims were to understand how stress physiology, measured at baseline and in response to restraint handling, and body condition changed over multiple days of the storm, and if these responses were consistent across years. Snowstorms did not affect baseline corticosterone concentrations for either species except for female Lapland longspurs and male white-crowned sparrows in 2014. Lapland longspurs, regardless of sex, increased stress-induced (restraint handling) corticosterone in response to snowstorms in all years but 2013, which was characterized by unusually harsh conditions. Both sexes of White-crowned sparrows showed a significant increase in the stress-induced levels of corticosterone during snowstorms in one of the three years of the study. Stress-induced corticosterone concentrations were only different across each day of the storm in one year of the study for Lapland longspurs. Changes in fat and body mass were not uniform across years, but measurable increases in fat stores and body mass were detected in males of both species during the first day of a snowstorm with declines typically occurring by the second day. Our study showed that severe weather events often caused rapid increases in HPA axis activity and body condition, but these profiles are likely dependent upon ecological and environmental context within the breeding season.

## Highlights

• Stress physiology was analyzed in response to multi-day snowstorms in 5 years.
• Baseline corticosterone was unaffected by snowstorms in all but two instances.
• Stress-induced corticosterone were typically elevated during snowstorms.
• Stress physiology was significantly different across a multiday storm in only one year.
• Body condition tended to increase on the first day of the storm.

## 1 Introduction

When migratory songbirds arrive on their breeding grounds they face many environmental challenges that vary from year to year such as predation, snow cover, severe weather, social instability, and food shortages (Newton, 2006, Wingfield et al., 2015). Each of these factors can cause physiological and/or psychological stress that disrupt homeostasis. Birds, like other vertebrates, rely upon the activation of the hypothalamic-pituitary adrenal (HPA) axis, including the production of glucocorticoids, to regulate physiology and behavior and restore homeostasis following an environmental perturbation (Sapolsky et al., 2000, Romero and Wingfield, 2015). Circulating levels of corticosterone can fluctuate between baseline and stress-induced levels, the latter often experimentally studied via restraint handling (Romero, 2002, Wingfield and Sapolsky, 2003). Baseline levels of corticosterone act primarily through high affinity mineralocorticoid receptors (MR), while stress-induced concentrations act on low affinity glucocorticoid receptors (GR) (Joëls et al., 2008, de Kloet, 2014). This creates a two-tier system of receptor activation by which physiology and behavior can be modified based on the level of HPA axis activation (Sapolsky et al., 2000, Angelier and Wingfield, 2013, de Kloet, 2014).

Studies across taxa have shown that HPA axis activity is highly plastic and is modulated according to life history stage (i.e., breeding, molt, migration, winter) (Astheimer et al., 1994, Romero et al., 1998b, Reneerkens et al., 2002, Romero, 2002, Holberton and Wingfield, 2003, Meddle et al., 2003, Krause et al., 2015b, Krause et al., 2015c, Walker et al., 2015). In many species, both baseline and stress-induced corticosterone concentrations are higher during the breeding season than any other time of the year (Romero, 2002). Taken together, these findings suggest an evolutionarily conserved process by which endocrine set points are adjusted throughout the annual cycle to meet energetic demands associated with each life history stage. For instance in birds, baseline levels of corticosterone likely vary in a nonlinear fashion with parental investment: moderate levels of baseline corticosterone increase parental care, while high levels reduce or terminate parental care (Wingfield et al., 1983, Raouf et al., 2006, Bonier et al., 2009, Spée et al., 2010, Ouyang et al., 2012, Thierry et al., 2013, Vitousek et al., 2014, Ouyang et al., 2015). Corticosterone has been proposed to be an important mediator of life history trade-offs between survival and reproduction because of this relationship (Wingfield et al., 1998). Prolonged elevation of corticosterone can cause the individual to redirect available energetic resources towards self-maintenance and abandon other activities such as breeding, a suite of responses termed the “emergency life history stage” (Wingfield et al., 1995, Wingfield et al., 1998, Angelier and Chastel, 2009). The threshold for triggering the emergency life history stage is thought to fluctuate across both seasonal activities and an individual’s life-time due to the associated fitness cost of abandoning the current life history stage (Wingfield and Kitaysky, 2002).

HPA axis activity, as measured through baseline and stress-induced corticosterone levels, has been shown to increase during inclement weather (Smith et al., 1994, Raouf et al., 2006, Krause et al., 2016a). For migrants and resident species breeding in the Arctic, snowstorms are a common type of severe weather event that can occur at any point during the summer. With climate change, severe weather events are occurring more often and are predicted to continue to increase in frequency (Smith, 2011, Wingfield et al., 2017). Snowstorms are energetically challenging as snow cover reduces access to food and low temperatures increase thermoregulatory costs (Kendeigh, 1969, Astheimer et al., 1995, Krause et al., 2016a). As a consequence, if negative energy balance occurs, fuel stores are mobilized to meet energetic demands (likely through corticosterone signaling), which results in a decrease in body condition (Landys et al., 2006, Krause et al., 2017). The influence of just a single storm event has been shown to elevate both baseline (a sample collected within three minutes of capture) (Wingfield et al., 1983, Smith et al., 1994, Astheimer et al., 1995, Jenni-Eiermann et al., 2008) and stress-induced levels (measure over a 1 h sampling period) (Krause et al., 2016a) of corticosterone. In addition, the duration of the storm (in days) may influence the magnitude of these changes (Wingfield, 1985b, Wingfield, 1985a, Angelier and Wingfield, 2013). Elevated corticosterone levels have been correlated with promoting escape behavior (Breuner and Hahn, 2003, Lynn et al., 2003) and foraging (Astheimer, Buttemer and Wingfield, 1992), as well as additional behavioral and physiological adjustments such as changes in antipredator behavior (Cockrem and Silverin, 2002, Jones et al., 2015) and immunological alterations (Sapolsky, Romero and Munck, 2000). Failure to respond appropriately to a stressor, in this instance a snowstorm, may result in decreased lifespan and/or reproductive output (McEwen and Wingfield, 2003, Angelier and Wingfield, 2013).

Previous studies on storm events have only described hormonal responses, typically baseline concentrations, during a single storm event (1 day) while not accounting for how physiology and body condition might be adjusted as the storm progresses across multiple days. This represents a serious gap in our understanding of how birds respond to unpredictable events and whether there is a unifying pattern of hormonal signaling and changes in body condition when the emergency life history stage is activated. We studied Lapland longspurs (*Calcarius lapponicus*) from 2012 to 2016 and Gambel’s white-crowned sparrows (*Zonotrichia leucophrys gambelii*) from 2014 to 2016 on their breeding grounds in the vicinity of Toolik Lake Research Station, in the Low Arctic tundra ecosystem of the North Slope of Alaska, USA. Both species have been well characterized with regards to their breeding biology and stress physiology in the Low Arctic and how they respond behaviorally and physiologically to both severe and benign conditions (Astheimer et al., 1995, Krause et al., 2015c, Krause et al., 2016a). Data collected at Toolik Lake indicate that average local spring temperatures have not changed in the last thirty years despite measurable changes at other arctic sites (Hobbie et al., 2017). However in the Toolik area thawing degree days have increased 23% over the past 50 years and peak stream discharge rates, which are often used as a proxy for spring snowmelt, indicate snowmelt is occurring earlier in the spring by 1.8 days per decade over the past 40 years (Tape et al., 2016). Other changes include the occurrence of snow fall in the spring appearing to be shifting later in the year indicated by a study in the Canadian Arctic (Nelson et al., 1997, Xiaogang et al., 2015). In each year of this study, snowstorms occurred in late May or early June, providing us with an unprecedented five years of data on Lapland longspurs and three years on white-crowned sparrows. The goals of this study were to understand 1) if physiology and body condition changed in response to severe snowstorms, 2) if these responses changed over the duration of the storm and immediately after its conclusion, 3) if the responses differed by sex and species, and 4) if these responses were repeatable at the population level across years. We predicted that both baseline and stress-induced corticosterone would be elevated during snowstorms, that body condition would decline over the duration of the storm, and that these responses would be repeatable across years. A basic understanding of all these factors will give us a mechanistic understanding of how organism will cope with a world that is experiencing an increase in extreme weather events.

## 2 Materials and methods

### 2.1 Study Site, Species, and phenology

This study was conducted from 2012 to 2016 during May and June at Toolik Lake Field Station (N 68° 38′, W 149° 36′), located in the foothills of the Brooks Range on the North Slope of Alaska, USA. Lapland longspurs and Gambel’s white-crowned sparrows are long-distance migratory songbirds that winter in the contiguous United States and breed at higher latitudes (Blanchard and Erickson, 1949, West et al., 1968). Lapland longspurs are arctic specialists that have a circumpolar breeding distribution and nest in tussock and polygon tundra (Boelman et al., 2014, Walker et al., 2015). Gambel’s white-crowned sparrows are thought to be more recent colonizers of the Low Arctic and primarily breed throughout the boreal forest (Krause et al., 2015a). In the Low Arctic, white-crowned sparrows prefer to nest on tundra dominated by deciduous woody shrubs and evergreens (Boelman et al., 2014, Boelman et al., 2016). Spring phenology of the breeding life history stage can be divided into sub-stages including arrival on breeding grounds, territoriality, clutch initiation and incubation and have been outlined in detail in Boelman et al. (2017). Both species typically arrive (“arrival period”) on the breeding grounds between the first and third week of May, depending on environmental conditions. Conditions upon arrival are characterized by extensive snow cover, patchy resource availability, low ambient temperatures, and reduced shelter (Custer and Pitelka, 1977, Boelman et al., 2017). Since neither species cache food they are dependent upon finding overwintered food sources from the previous summer that are not covered by snow (Wingfield et al., 2004). Both species establish territories (“territorial period”) once environmental conditions become conducive for breeding, as snow melts, temperatures warm, and food resources increase. The termination of the territory acquisition period is marked by clutch initiation (“clutch initiation period”) when the first egg is laid, which typically commences in the last week of May through the first week of June for both species (Boelman et al., 2017). Once the last egg is laid females begin incubating the eggs (“incubation period”) for a period of 12 days (Custer and Pitelka, 1977). Due to the shortness of the arctic summer, both of these species have a brief window of opportunity in which they can breed (Oakeson, 1954, Custer and Pitelka, 1977, Boelman et al., 2017) and snowstorms or other major environmental perturbations can greatly disrupt reproductive behavior (Astheimer et al., 1995, Krause et al., 2016a, Pérez et al., 2016).

Study sites were observed intensively for nesting females to locate nests; clutch initiation dates were estimated by the last day in which an egg was laid with the assumption that 1 egg was laid per day and backdated based on the number of eggs; if clutch was complete, nests were checked daily till hatching to determine daily survival and backdated using mean incubation duration with the assumption of 1 egg laid per day with incubation beginning with the last egg.

## 3 Weather and snow cover

Snowstorms were defined as snowfall that resulted in detectable increases in snow cover within a single 24 h period. Weather data including temperature, precipitation (mm), barometric pressure (kPa), relative humidity (%), and wind speed (m/s) were collected by meteorological stations positioned 3 m above the ground during the respective periods in which birds were sampled (Toolik Environmental Data Center, 2017). Time lapse photography was employed to assess snow cover by collecting images at 1200 h from a stationary tripod. Percent snow cover captured by each image was quantified using ImageJ (NIH, Bethesda, MD). The threshold tool was used by first converting the image to 8 bit color, then white pixels were converted to red pixels, and the number of red pixels in the image was quantified to produce % snow-covered ground. Snow cover values are reported as the daily mean of the three photos (Krause et al., 2016a).

## 4 Capture and blood sampling

We captured a total of 420 Lapland longspurs and 297 white-crowned sparrows to assess body condition. Of these we sampled blood for corticosterone analyses from a total of 116 male and 88 female Lapland longspurs between 2012 and 2016 and 81 male and 30 female white-crowned sparrows from 2014 to 2016. Sampling occurred between 08:30 and 16:00 h and previous data show that time of day has no effect on corticosterone during these hours (Krause et al., 2015c). Unique individuals were divided into those caught on storm-free days, those caught during snowstorms, with the latter further subdivided into the day (1–3) of the snowstorm, and 1 day post snowstorm. During the early breeding season prior to territory establishment, both species were caught with seed-baited potter traps. Once birds were territorial, white-crowned sparrows were caught almost exclusively with Japanese mist nets, while Lapland longspurs were caught with both potter traps and mist nets. Birds were caught exclusively with potter traps during snowstorms.

The alar vein was punctured with a 26 gauge needle and a baseline blood sample (40 μL) was collected into a heparinized microcapillary tube within 3 min of capture. On average, samples were collected within 118 ± 32 (S.D.) seconds of the bird entering the trap or making contact with the mist net; this should reflect baseline or near baseline hormone concentrations in both of these species (Romero and Reed, 2005). We used previously described restraint protocols to measure HPA axis activity in response to acute stress (Krause et al., 2015a). Briefly, blood samples were collected at 10, 30 and 60 min post-capture and birds were held in an opaque cloth bag in between samples. Unique leg color band combinations and an aluminum numbered band were given to each bird for later identification in the field. Morphometrics of wing chord, tarsus and beak were measured with calipers to the nearest 0.1 mm, body mass was measured with a Pesola Scale to the nearest 0.5 g, and fat stores (furcular and abdominal) were scored on a scale from 0 (lean) – 5 (fat) (Kaiser, 1993). Blood samples were stored on ice until later processing in the laboratory. Samples were centrifuged at 13,000*g* for 5 min to separate erythrocytes from plasma. The plasma was aspirated, placed it into a microcentrifuge tube, and stored at −80 °C until hormone quantification.

## 5 Corticosterone assay

Corticosterone levels were quantified using a radioimmunoassay as previously described in detail by Krause et al. (2015a). In brief, 15 μL of plasma was combined with 2,000 CPM of tritiated corticosterone to determine extraction efficiency for each sample. Each sample was mixed with 4 mL of redistilled dichloromethane for 3 h to extract steroids from plasma. Extracts were dried under nitrogen at 35 °C, and reconstituted in 550 µL phosphate-buffered saline with gelatin (PBSG). A 100 µL aliquot was added to a scintillation vial and combined with scintillation fluid (Perkin Elmer Ultima Gold: 6013329) to determine percent recoveries. Duplicate 200 µL aliquots were assayed by adding 100 µL (∼10^4^ CPM) of tritiated corticosterone (Perkin Elmer NET399250UC) and 100 µL of antibody. (Samples from 2012 to 2015 were run with Esoterix Inc. B3-163 antibody, and samples from 2016 with MP Biomedical 07–120016, lot 3R3-PB-20E antibody). Unbound was separated from bound steroid with 500 µL of dextran-coated charcoal followed by centrifugation at 3000*g*. The supernatant was decanted and combined with scintillation fluid and counted for 5 min or within 2% accuracy on a Beckman 6500 LS counter. Results were averaged across duplicates and corrected for individual sample recoveries. Mean recoveries were 85.78% and intra-assay (calculated using C.V. between duplicates) and inter-assay variations were 7.25% and 10.87%, respectively. The mean ± standard error for the detection limits of the assays was 8.87 ± 0.49 pg per tube (∼0.7 ng/mL per tube).

## 6 Statistical analyses

Statistical analyses were performed using JMP 12 Pro (SAS Institute Inc., Cary, NC, 1989-2007). All data were checked for normality using the Shapiro-Wilks test by plotting the residuals against the predicted value. For all analyses we were interested in how the response variable changed across the days of the storm. As such, we coded each day of the storm as day 1, day 2 and day 3. Three of the four years of the study had 1–2 day snowstorms; only 2014 had a 3 day snowstorm. To compare differences in weather variables between storm and storm-free days, separate analysis of covariance (ANCOVA) with day-of-year as a covariate were performed for mean temperature, min temperature, max temperature, total precipitation, wind speed (m/s), max wind speed, barometric pressure (kPa), and percent snow cover.

We compared stress physiology and body condition data that were collected during the closest sub-stages of breeding that occurred during the storms (i.e. arrival period/territorial period, and egg laying/ incubation period as described in Table 1) within a given year. This approach allowed us to control for the effects of life history stage as best as possible given the distribution of our sampling. To test if there was a typical pattern in which birds responded to snowstorms, the overall patterns of hormonal response to stress, body mass and fat score from all years were combined to test how each response variable was affected by sex, stress, day of storm and their interactions while individual was included as random effect nested within year and life history stage. For each year of the study, a mixed effects model with individual as a random effect and a residual covariance structure was used to test how corticosterone levels were affected by sex, stress (sampling time point), day of storm and their interactions. Fat scores were analyzed using a logistical fit model to test for differences between days with or without snowstorms. Body mass was analyzed for each sex using a linear effects model with the main effect of storm. The effect of snowstorms on body condition was analyzed using a one-way ANOVA. All post hoc analyses were performed using Tukey’s Honestly Significant Difference (HSD) test and the associated t-tests are reported. All values are presented as means ± SEM.

**Table 1:** A summary of the days on which storms occurred in each year, substage of breeding at the time of the storm, the percent of nests that failed because of the storm, and whether or not stress-induced corticosterone were affected by the snowstorm. Clutch initiation dates and substages are taken from Boelman et al. (2017).

Year	2012	2013	2014	2015	2016
*Lapland longspurs*					
Storm dates birds sampled	May 26–27	June 6–7	May 17, 18, & 25	May 31, June 2 & 11	June 5, 8, & 21
Life history stage during storm(s)	Territorial/Laying	Laying	Territorial	Laying & incubation	Incubation
Mean clutch initiation date	May-30	Jun-04	May-30	NA	May-31
Nest failure during storm %	NA	16	5	NA	90
Stress affected by storm	Yes	No	Yes	Yes	Yes

*White-crowned sparrows*					
Storm dates birds sampled	NA	NA	May 13, 18, 22, & 25	June 2 & 11	May 24–25, June 5 & 8
Life history stage during storm(s)	NA	NA	Territorial	Incubation	Territorial & incubation
Mean clutch initiation date	May-31	Jun-04	Jun-01	May-25	Jun-01
Nest failure during storm %	NA	NA	9	30	80
Stress affected by storm	NA	NA	No	No	Yes

## 7 Results

### 7.1 Snowstorms and bird phenology

**Fig. 1:**
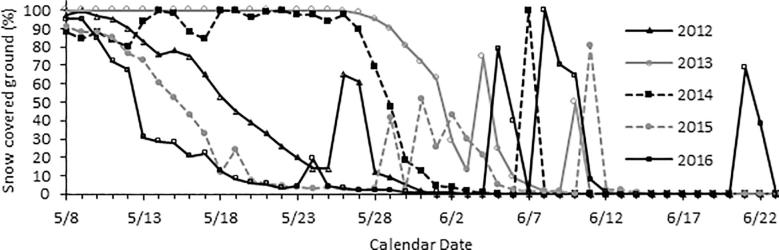
Changes in percent snow cover over the course of the breeding season at Toolik Lake Alaska for the five study years (2012–2016). Snowstorms occurred in every year of the study and can be seen as the rapid increases in snow cover.

Seasonal declines in snow cover often occurred rapidly in spring with sharp increases occurring during snowstorms (Fig. 1). Timing, intensity, and duration of snowstorms varied greatly over the 5 years of the study. The greatest precipitation and change in snow cover occurred on the first day of the snowstorm (Table 2). Snow cover ranged from 60 to 100% on days in which snowstorms occurred, compared to 0–90% on storm-free days depending upon the sampling point within a given year (Fig. 1). Snow fall often declined in the afternoon prior to increasing again in the evening which allowed for a certain amount of melt to occur as reflected in percent snow cover measurements that were below 100% (Krause personal observations).

**Table 2:** A comparison of environmental parameters during snowstorms and compared to snowstorm free days. Each parameter was tested using an ANCOVA to control for day of year.

Variable	Storm-free	Day 1	Day 2	Day 3	1 Day post	*D.F.*	*F*	*P*
Mean air temperature (°C)	5.33 ± 0.22	−1.64 ± 0.32	−0.62 ± 0.10	−6.51	−1.16	**1,114**	**536.99**	**<0.001**
Min air temperature (°C)	−0.49 ± 0.16	−4.33 ± 0.41	−5.97 ± 0.06	−8.81	−4.84	**1,114**	**628.6**	**<0.001**
Max air temperature (°C)	9.58 ± 0.26	2.01 ± 0.50	5.27 ± 0.44	5.17	5.00	1,114	1.74	0.18
Mean daily wind speed (m/s)	2.55 ± 0.07	3.34 ± 0.09	2.41 ± 0.03	2.89	1.63	**1,114**	**71.87**	**<0.001**
Max wind speed (m/s)	5.74 ± 0.22	7.01 ± 0.39	5.20 ± 0.11	5.17	4.20	**1,114**	**4.85**	**0.02**
Relative humidity (%)	73.15 ± 1.10	89.97 ± 0.19	88.82 ± 0.53	88.89	79.58	**1,114**	**130.02**	**<0.001**
Snow Cover (%)	12.81 ± 2.24	66.25 ± 0.36	18.64 ± 3.23	91.70	40.01	**1,114**	**291.42**	**<0.001**
Air pressure (kPa)	93.84 ± 0.11	91.39 ± 0.12	92.59 ± 0.07	92.63	92.40	**1,114**	**12.17**	**<0.001**
Total precipitation (cm)	0.02 ± 0.005	0.14 ± 0.01	0.05 ± 0.005	0.05	0.03	1,114	34.06	**<0.001**

In 2012, snow melt occurred early in the season and progressed rapidly (Fig. 1). From May 26 to 27 there was a heavy snowstorm at a time when females were just starting to lay eggs. In contrast 2013 was characterized by an unusual persistence of snow cover into the late spring (Fig. 1). A snowstorm occurred on June 6 and 7 during the transition from nest building to egg laying in 2013 (Table 1). In 2014, snow cover was low in the early season but repeated snowstorms on May 13, 17, 18, 21, 22, 23, 25 increased snow cover to nearly 100% over much of the in the Toolik region and a final snowstorm occurred on June 7 during incubation. The 2015 field season was characterized by unusually early snow melt. The first snowstorm occurred during May 29–31 when birds were laying eggs, a second on June 2 and a third on June 11 during incubation (Table 1). Finally, 2016 had early snow melt that occurred on a similar trajectory to 2015. Snowstorms in 2016 occurred on May 24 and 25 when birds were territorial and again on June 5, 8, and 21 during the incubation period. A summary of snowstorm dates, life history stages at the time of the snowstorm, and mean clutch initiation dates taken from can be found in Table 1.

#### 7.1.1 Meteorological data

During snowstorms, air pressure, mean and minimum air temperatures were significantly lower; and relative humidity, snow cover, mean and max wind speeds, and precipitation were higher than on storm-free days (Table 2 and Fig. 1). Maximum air temperature was not different between storm-free days and snowstorm days (Table 2).

#### 7.1.2 HPA axis activity in Lapland longspurs

**Fig. 2:**
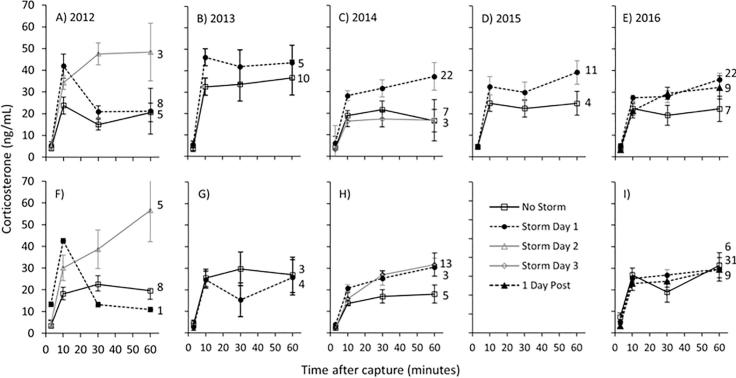
The effect of snowstorms on plasma corticosterone levels in response to acute restraint stress in Lapland longspur males (A,B,C,D,E) and females (F,G,H,I) in five separate years from 2012 to 2016. For each year, males and females are aligned in the same column of graphs except for 2015 in which there was an insufficient sample size for females. There was a significant main effect of snowstorms on corticosterone concentrations in all years except 2013. There was a significant interaction between day of storm and stress in 2012, only. Samples sizes for each group are indicated next to the 60 min time point. Values are presented as mean ± SEM.

Under all environmental conditions, acute restraint stress resulted in increased corticosterone concentrations and no differences were detected between the sexes (Table 3). When all years were combined in an analysis, the main effects of day of storm (F_3, 19_ = 16.06, *P* < 0.001), restraint stress (F_3,593_ = 90.74, *P* < 0.001, and the interaction of day of storm and restraint stress (F_9,593_ = 5.91, *P* < 0.001) while sex along with all of its interactions were not significant and were removed from the model. Each day of the storm was significantly different with the highest levels observed on day 2, then lower on day 1, while no differences were found between day 3 and storm-free conditions. Analysis of individual years indicated that stress-induced corticosterone concentrations were significantly higher during snowstorms compared to storm-free days in 2012, 2014, 2015, and 2016, but not 2013 (Table 3). Stress-induced levels of corticosterone on storm-free days were significantly higher in 2013 compared to all other years. The pattern of corticosterone synthesis across the days of the snowstorm was significantly different in 2012 only, as indicated by the interaction between day of snowstorm and stress (sampling time point; Fig. 2a and f). Post hoc analyses of the 2012 data indicated that corticosterone was significantly elevated at the 10 min sampling point on both day 1 (t = 3.91, *P* < 0.001) and day 2 (t = 3.01, *P* < 0.001) of the snowstorm compared to storm-free days. On day 2 of the snowstorm, corticosterone at both the 30 (t = 6.10, *P* < 0.001) and 60 (t = 3.89, *P* < 0.001) minute time points was also significantly higher than on storm-free days. In females, baseline concentrations of corticosterone were significantly higher on day 1 of the snowstorm compared to storm-free days in 2014 (F_2,18_ = 4.01, *P* = 0.03; Fig. 2h and Supp. Fig. 1). No other significant differences were found in baseline concentrations of corticosterone in for either sex (Supp. Fig. 1).

**Table 3:** The effects of snowstorms, sex, and acute restraint stress (stress) across five separate years in Lapland longspurs. Main effects and their interactions were tested using a linear mixed effects model in individual included as a random factor.

Independent variable	2012				2013				2014				2015				2016			
	*D.F.*	*F*	*P*		*D.F.*	*F*	*P*		*D.F.*	*F*	*P*		*D.F.*	*F*	*P*		*D.F.*	*F*	*P*	
Sex	1,25	0.01	0.92		**1,19**	**4.80**	**0.04**		1,40	0.75	0.39		NA	NA	NA		1102	0.22	0.63	
Storm	2,25	**7.46**	**0.003**		1,22	0.15	0.69		**2,40**	**9.40**	**<0.0001**		**1,10**	**4.86**	**0.05**		**2122**	**3.15**	**0.04**	
Stress	3,75	**54.29**	**<0.0001**		**3,54**	**17.92**	**<0.0001**		**3130**	**168.71**	**<0.0001**		**3,33**	**17.50**	**<0.001**		**3295**	**65.52**	**<0.001**	
Sex * Storm	2,25	0.03	0.97		1,22	1.83	0.18		2,40	1.65	0.20		NA	NA	NA		2122	0.09	0.91	
Sex * Stress	3,75	0.70	0.56		3,54	1.16	0.33		3130	2.18	0.09		NA	NA	NA		3295	0.87	0.45	
Storm * Stress	6,75	**5.29**	**<0.0001**		3,54	0.58	0.62		6130	0.49	0.81		3,33	1.45	0.24		6295	1.47	0.18	
Sex * Storm * Stress	6,75	1.75	0.12		3,54	0.25	0.85		6130	0.69	0.65		NA	NA	NA		6295	0.42	0.86

#### 7.1.3 HPA axis activity in white-crowned sparrows

**Fig. 3:**
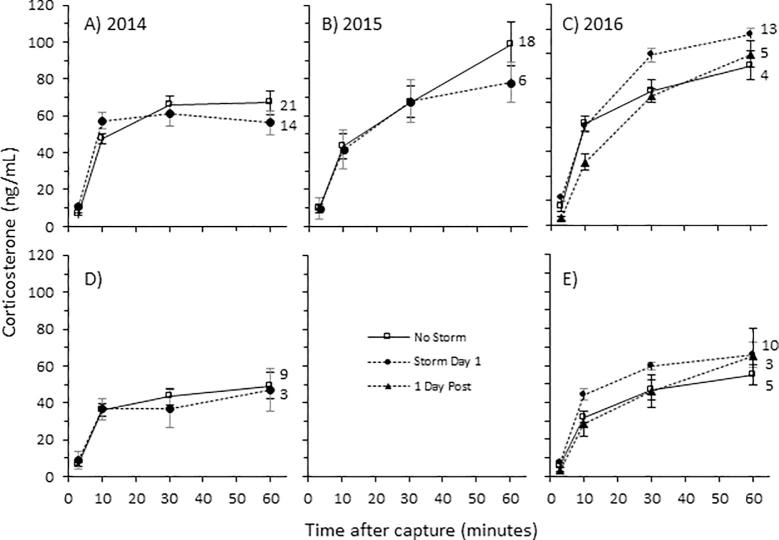
The effect of snowstorms on plasma corticosterone concentrations in response to acute restraint stress in (A,B,C) male and (D,E) female white-crowned sparrows in three separate years 2014–2016. For each year, males and females are aligned in the same column of graphs except for 2015 in which there was an insufficient sample size for females. There was a significant main effect of snowstorms on corticosterone concentrations in 2016 which was primarily driven by the males’ response to snowstorms. Samples sizes for each group are indicated next to the 60 min time point. Values are presented as mean ± SEM.

Corticosterone increased in response to acute restraint stress in white-crowned sparrows regardless of the weather (Table 4 and Fig. 3). When all years were combined in an analysis, the main effects of sex (F_1,96_ = 6.2, *P* = 0.01), restraint stress (F_3,311_=, *P* < 0.001), and the interaction of sex and restraint stress (F_3,311_ = 2.68, *P* = 0.04) were significant while the main effect of day of storm (F_1,108_ = 0.12, *P* = 0.72) and all of its interactions were not significant. Analysis of individual years indicated that in both sexes stress-induced corticosterone concentrations were higher on snowstorm days compared to storm-free days in 2016 while this effect was absent in 2014 and 2015 (Table 4). Corticosterone concentrations during acute stress on storm-free days were higher in males than females and were significantly different at the 10 (t = 7.39, *P* = 0.04), 30 (t = 2.93, *P* = 0.007) and 60 min time points (t = 4.84, *P* < 0.001 In males, baseline concentrations of corticosterone were significantly higher on day 1 of the snowstorm compared to storm-free days in 2014 (F_1,35_ = 7.22, *P* = 0.01; Fig. 3a and Supp. Fig. 2) while no other differences were detected

**Table 4:** The effects of snowstorms, sex, and acute restraint stress (stress) across three separate years in white-crowned sparrows. Main effects and their interactions were tested using a linear mixed effects model in individual included as a random factor.

Independent variable	2014				2015				2016		
	*D.F.*	*F*	*P*		*D.F.*	*F*	*P*		*D.F.*	*F*	*P*
Sex	**1,45**	**7.09**	**0.011**		NA	NA	NA		1,57	0.742	0.39
Storm	1,45	0.06	0.81		1,83	2.93	0.09		**2,69**	**6.187**	**0.003**
Stress	**3135**	**40.44**	**<0.001**		**3,70**	**57.41**	**<0.001**		**3130**	**65.0**	**<0.0001**
Sex * Storm	1,45	0.45	0.506		NA	NA	NA		2,69	0.436	0.648
Sex * Stress	3135	0.32	0.81		NA	NA	NA		3130	0.279	0.84
Storm * Stress	3135	1.98	0.12		3,70	0.91	0.43		3130	0.219	0.97
Sex * Storm * Stress	3135	0.25	0.86		NA	NA	NA		6130	0.314	0.93

#### 7.1.4 Body condition in Lapland longspurs

When all years were combined mass was not affected by day of storm (F_3,273_ = 1.16, *P* = 0.32) but were different by sex (F_1,446_ = 21.78, *P* < 0.01) and by the interaction of sex and day of storm (F_3,373_ = 3.81, *P* = 0.01). However, post hoc tests did not indicate a that body mass for either sex was directly influenced by snowstorms but rather differences across days of the storm between males and female (Tukey’s HSD *P* < 0.05). Similarly, when all years were investigated fat scores were not directly influenced by day of storm (F_3,237_ = 1.44, *P* = 0.22) or the interaction of sex and day of storm (F_3,443_ = 1.56, *P =* 0.19) but was affected by sex (F_1,472_ = 4.36, *P* = 0.03) with females having higher fat scores overall.

**Fig. 4:**
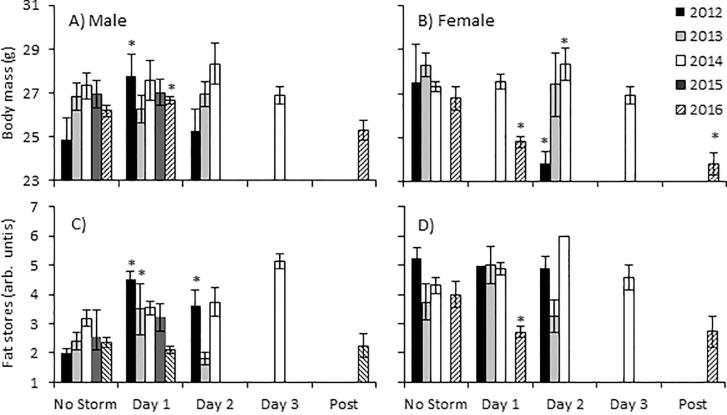
The effect of snowstorms on body mass and fat in (A,C) male and (B,D) female Lapland longspurs. Sample sizes can be found in Table 5. Values are presented as mean ± SEM. Asterisks indicates significance of P < 0.05 compared to storm-free days.

When comparing body mass and fat stores within years, they were not consistently predicted by day of storm for either sex (Table 5 and Fig. 4). A detectable increase in body mass was found for males in 2012 (t = 2.87, *P* = 0.008) and 2016 (*t = 3.19, P = 0.003*) during day 1 and for females in 2014 on day 2 of the storm compared to storm-free days, while in 2016 body mass declined in response to day 1 of the storm in females (t = 3.01, *P* = 0.002) and remained low 1 day post storm (t = 3.14 , *P* = 0.001). Fat scores increased in response to snowstorms in males in 2012 and 2013 while they declined in 2016 (Table 5, Fig. 4). In 2012, fat stores in males were significantly higher on day 1 (χ^2^ = 12.36, *P* = 0.01) and day 2 (χ^2^ = 13.14, *P* = 0.04) of the storm compared to storm-free days. In 2013, male fat stores were significantly higher on day 1 (χ^2^ = 10.02, *P* = 0.04) but were not different on day 2 (χ^2^ = 5.71, *P* = 0.12) of the storm compared to storm-free days in 2013. Female fat stores declined on day 1 of the snowstorm in 2016 (*χ*^2^ = 6.91, *P* = 0.008).

**Table 5:** The effect of snowstorms on total fat and body mass in male and female Lapland longspurs and white-crowned sparrows. The effects of snowstorms on Fat measures were tested using an ordinal logistic model while body mass was tested using a linear mixed effects model.

Year	Sex	Lapland longspur						White-crowned sparrow					
		Total Fat			Body Mass			Total Fat			Body Mass		
		*n*	*χ*^2^	*P*	*D.F.*	*F*	*P*	*n*	*χ*^2^	*P*	*D.F.*	*F*	*P*
2012	Males	20	**18.10**	0.05*	**2,19**	**5.46**	0.01*	0	NA	NA	NA	NA	NA
	Females	14	2.80	0.24	**1,12**	**7.23**	0.02*	0	NA	NA	NA	NA	NA
2013	Males	19	**15.73**	0.04*	2,18	0.22	0.80	0	NA	NA	NA	NA	NA
	Female	12	2.72	0.25	1,11	0.5	0.49	0	NA	NA	NA	NA	NA
2014	Males	38	20.00	0.14	3,77	1.06	0.37	100	**33.32**	0.002*	**2,97**	**4.68**	0.01*
	Female	32	4.84	0.16	**3,31**	**8.93**	0.009*	26	11.70	0.30	2,23	1.82	0.18
2015	Males	24	3.65	0.72	1,23	0.006	0.93	31	1.18	0.27	1,29	0.01	0.89
	Female	0	NA	NA	NA	NA	NA	28	0.02	0.88	1,24	0.45	0.51
2016	Males	166	0.99	0.60	**2,160**	**4.6**	0.01*	70	5.27	0.07	2,66	0.39	0.67
	Female	**95**	**7.08**	0.02*	**2,92**	**7.38**	0.001*	42	4.99	0.08	2,38	0.83	0.44

#### 7.1.5 Body condition in white-crowned sparrows

When all years were combined mass was affected by sex (F_1,247_ = 8.08, *P* = 0.004) but not by day of storm (F_4,188_ = 0.58, *P* = 0.56) or the interaction of sex and day of storm (F_2,187_ = 0.25, *P* = 0.77). Fat scores combined across years were affected by sex (F_1,301_ = 6.93, *P* = 0.008), storm (F_3,283_ = 2.79, *P* = 0.04 and the interaction of storm and sex (F_3,297_ = 3.01, *P* = 0.01). Post hoc analysis indicated that fat scores in females were lower on day 1 and one day post storm compared to storm free-days while males were unaffected.

**Fig. 5:**
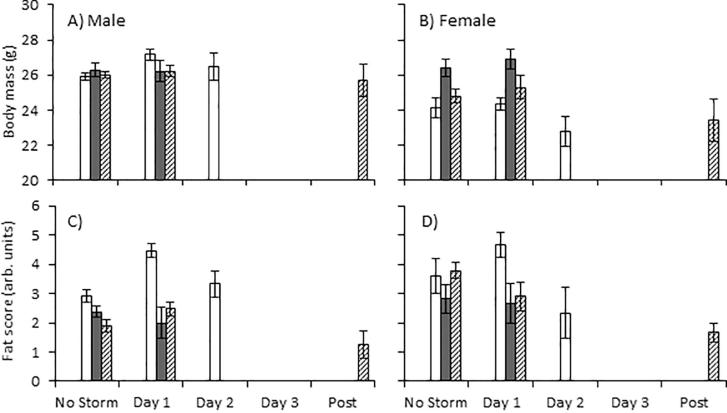
The effect of snowstorms on body mass and fat in (A,C) male and (B,D) female white-crowned sparrows. Sample sizes can be found in Table 5. Values are presented as mean ± SEM. Asterisks indicates significance of P < 0.05 compared to storm-free days.

Comparing fat scores within individual years, male white-crowned sparrow body mass and fat concentrations were affected by snowstorms only in 2014 while no other differences in body condition measurements were detected for either sex (Table 5 and Fig. 5). In males, during 2014, on day 1 of the snowstorm, body mass (t = 3.26, *P* = 0.004) and fat stores (χ^2^ = 19.97, *P* = 0.005) were higher compared to storm-free days. By day 2 of the snowstorm body mass (t = 0.88, *P* = 0.65) and fat stores (χ^2^ = 8.77, *P* = 0.26) were not different from storm-free days.

## 8 Discussion

Snowstorms resulted in challenging environmental conditions that featured lower temperatures, increased wind speed, and greater precipitation and snow cover than snow-free days. In agreement with our hypothesis, HPA axis activity was predominantly higher on days with snowstorms compared to those without for Lapland longspurs while this was less supported for white-crowned sparrows. In four out of five years, Lapland longspurs regardless of sex, had higher stress-induced corticosterone concentrations during snowstorms. This relationship was not observed in the extreme weather year (2013), which was characterized by unusually late snow melt, cold temperatures and delayed spring migration (Krause et al., 2016b, Boelman et al., 2017). In addition, stress-induced levels of corticosterone in Lapland longspurs were approximately double the concentrations in 2013 compared to other years (Krause et al., 2016b). Taken together, this suggests that HPA axis activity was increased throughout the 2013 breeding season, or perhaps even maximally increased such that it was similar to corticosterone values measured during snowstorms. This increase in HPA activity may compensate for the generally harsher conditions that year compared to all other years. In 2012, it was interesting to observe a measurable difference in HPA axis activity across each day of the snowstorm in Lapland longspurs, which was not observed in any other year. It is possible that the unique set of environmental conditions, timing of the snowstorm within the breeding season, or the number of storms experienced in each year results in stress profiles that were uniquely different across years. Elevated HPA axis activity by day 2 of the storm in 2012 may indicate a cost incurred over the duration of the snowstorm as birds are forced to draw on resources and increase foraging in an effort to maintain body condition.

In white-crowned sparrows, HPA axis activity as measured by stress-induced concentrations was increased by snowstorms only in 2016. Contrary to our prediction, HPA axis activity was not affected in every single year. White-crowned sparrow HPA axis activity shows greater seasonal modulation compared to Lapland longspurs breeding in the vicinity of Toolik Lake (Astheimer et al., 1994, Krause et al., 2015c). Since the stress response is so variable across the breeding season, sampling points that are too temporally spaced (i.e. even within two substages of breeding) can make it difficult to detect significant changes in corticosterone levels in response to snowstorms. For instance, when white-crowned sparrows first arrive on the breeding grounds the activity of the HPA axis is lower (compared to egg laying) despite environmental conditions being harsher (Krause unpublished data). The elevation in stress-induced corticosterone concentrations in males around the time of egg laying may suggest that the act of courting, mate guarding, and copulating results in heightened sensitivity to a stressor that may be less sensitive to further changes in environmental conditions. Sample points during storms in 2014 and 2015 occurred earlier in the breeding season during egg laying when HPA activity was higher compared to 2016 when incubation was well underway. Data from Lapland longspurs suggests that timing of the storm during reproduction had implications for the overall profile of the stress response (Krause et al., 2016a). Indeed at lower latitudes HPA axis activity in male Gambel’s and Puget Sound white-crowned sparrows were elevated either when breeding in harsher conditions or in response to severe weather events (Wingfield et al., 1983, Addis et al., 2011, Krause et al., 2015a). Higher corticosterone concentrations in birds breeding in a harsher environment may indicate that a unique set of physiological traits are important for survival (Wingfield et al., 2015).

The unique and repeatable patterns of plasma corticosterone during snowstorms in Lapland longspurs and white-crowned sparrows would suggest that this is a patterned response to an environmental perturbation and not the consequence of a single stochastic event. Rapid activation of the HPA axis in response to challenging events is consistent with previous work in Lapland longspurs and white-crowned sparrows breeding in extreme conditions at the northern extent of their range (Krause et al., 2015a, Walker et al., 2015). For Lapland longspurs, the level at which this rapid regulation occurs remains largely unknown as systemic injections of HPA axis agonists such as corticotropin releasing hormone (CRH), arginine vasotocin (AVT) and adrenocorticotropic hormone (ACTH) were unable to further increase circulating concentrations of corticosterone during the breeding season (Romero, Soma and Wingfield, 1998a). In a previous study on white-crowned sparrows sampled around the same time as Lapland longspurs, exogenous administration of these same agonists resulted in elevations in corticosterone concentrations beyond those elicited by Ringer’s solution alone (Romero and Wingfield, 1998). This suggests that the species which should be more sensitive to increased HPA activity does not appear to as readily modify HPA axis in response to environmental stressors (snowstorms), while Lapland longspurs which show no responsiveness to pharmacological stimulation of the HPA axis readily respond strongly to environmental stressors. Our current study suggests that HPA axis activity can be rapidly modulated in response to an environmental perturbation, although the exact mechanisms by which the hormonal endpoints for each species are achieved remain unknown.

Baseline concentrations of corticosterone regulate metabolic processes (Sapolsky et al., 2000, Landys et al., 2006). Baseline corticosterone concentrations in both species are highest during breeding when compared to wintering or molt which may help to meet increased metabolic demands associated with breeding (Astheimer et al., 1995, Krause et al., 2014). We predicted that baseline corticosterone would be increased during snowstorms in order to mobilize metabolic stores. We found that baseline corticosterone levels were elevated during snowstorms in Lapland longspur females and male white-crowned sparrows during 2014 season, but no differences were seen in other years. This suggests that birds were likely able to meet metabolic needs during snowstorms most years despite reduced access to foraging areas caused by increased snow cover. During the early season songbird diets are dominated by berries, seeds, and insect larvae (Custer and Pitelka, 1978, Norment and Fuller, 1997). Thus birds must work to uncover food resources buried by snow or even may be unable to access food sources (Wingfield et al., 2004) which increases energy expenditure along with the increased thermoregulatory costs associated with lower temperatures (Kendeigh, 1969, Custer et al., 1986). However, continuous snow fall throughout the entire day is often rare as pauses in snowfall typically occur (Krause personal observation). Partial melting of recently fallen snow during these pauses may have afforded sufficient time in which birds were able to forage and replenish fuel stores. This is partially supported by our finding of increased body condition metrics during the first day of the storm. Direct links between food intake and baseline corticosterone have been demonstrated in laboratory studies from white-crowned sparrows, house sparrows (*Passer domesticus*), and zebra finches (*Taeniopygia guttata*) using short-term removal or reductions in food intake to induced rapid elevations in baseline and stress-induced concentrations of corticosterone (Lynn et al., 2003, Lendvai et al., 2014, Krause et al., 2017). In the wild, severe weather events have been shown to elevate baseline concentrations of corticosterone (Wingfield et al., 1983, Wingfield, 1985b, Wingfield, 1985a, Rogers et al., 1993, Smith et al., 1994, Raouf et al., 2006, Jenni-Eiermann et al., 2008). Since body condition in general was not negatively affected by snowstorms it might not be surprising that we did not observe a consistent elevation in baseline corticosterone. It is also difficult to parse the effects of using seed baited potter traps on baseline corticosterone concentrations during the storm which may have had an effect as well.

In response to snowstorms, we predicted that body mass and fat scores would decline in both species in this study. Previous studies have demonstrated that in response to severe weather events body mass and fat stores often are reduced e.g. in Puget Sound white-crowned sparrows (*Z.l. pugetensis*), Lapland longspurs, Gambel’s white-crowned sparrow, white-tailed ptarmigan (*Lagopus leucura*) and song sparrows (*Melospiza melodia*) (Wingfield et al., 1983, Wingfield, 1985b, Wingfield, 1985a, Martin and Wiebe, 2004, Krause et al., 2016b). Counter to our prediction, there were inconsistencies in how mass and fat responded to snowstorms. The most common pattern was for body mass and fat stores to increase on the first day of the snowstorm and decline by the second day. This suggests that either that birds became hyperphagic prior to the arrival of, or during, the snowstorm as they fed on available food patches. Birds are able to detect changes in barometric pressure and may be able to predict an approaching storm front and prepare for it by increasing food consumption. No differences in body condition were detected for females but they tend to maintain higher fat levels likely so that they can weather a snowstorm during incubation. We also emphasize that each snowstorm presented unique challenges as each occurred at different times based on bird phenology and in relation to the phenology of their food for both species.

## 9 Conclusion

The intensity and frequency of severe weather events has been increasing worldwide as a consequence of climate change. Birds may rely on the HPA axis to adjust physiology and behavior in response to these unpredictable events. Our study showed that within a single life history stage, severe events can cause rapid increases in HPA axis activity in Lapland longspurs and white-crowned sparrows. However, hormonal profiles can vary by year depending on ecological and environmental context. The responses to severe events were repeatable across years suggesting that this response is typical and that it is likely adaptive for helping organisms cope with perturbations to their environment. In addition, fat stores and body mass in males tend to increase on the first day of the storm suggesting that hyperphagia must be occurring for there to be such rapid changes in body composition.

## 10 Author contributions

LG, JCW and NB conceived the ideas and designed methodology. JSK, JHP, HEC, KEH, and SLM collected the data. JSK analyzed the data. JSK led the writing of the manuscript. All authors provided feedback on manuscript.
