# Supplementary figures and images for: Weathering the storm: Do arctic blizzards cause repeatable changes in stress physiology and body condition in breeding songbirds?
Source: Gen Comp Endocrinol. 2018 Oct 1;267:183–92. doi: 10.1016/j.ygcen.2018.07.004 (PMC6127033; doi:10.1016/j.ygcen.2018.07.004)

## Slide 1
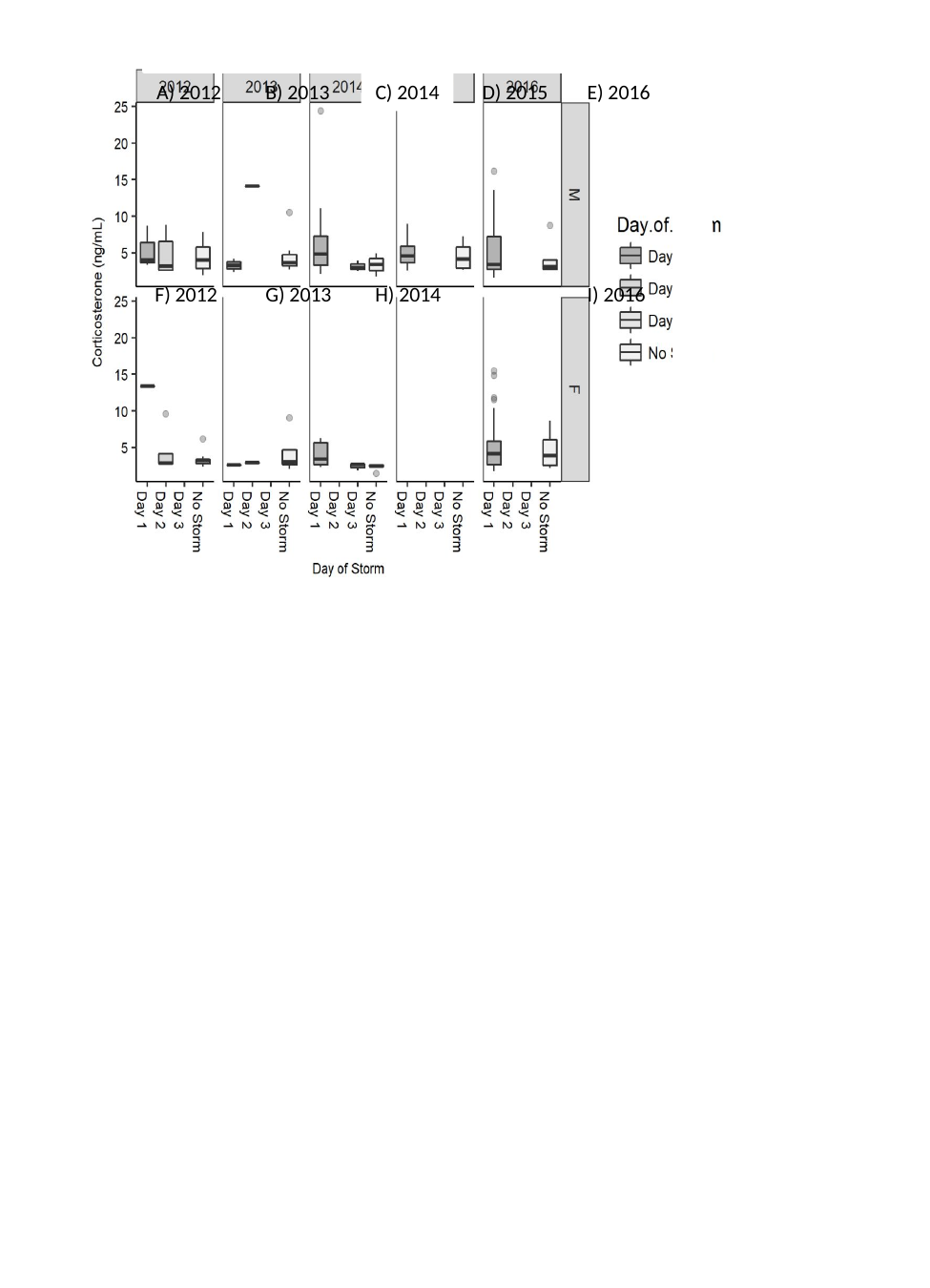

A) 2012
B) 2013
C) 2014
D) 2015
E) 2016
F) 2012
G) 2013
H) 2014
I) 2016

## Slide 2
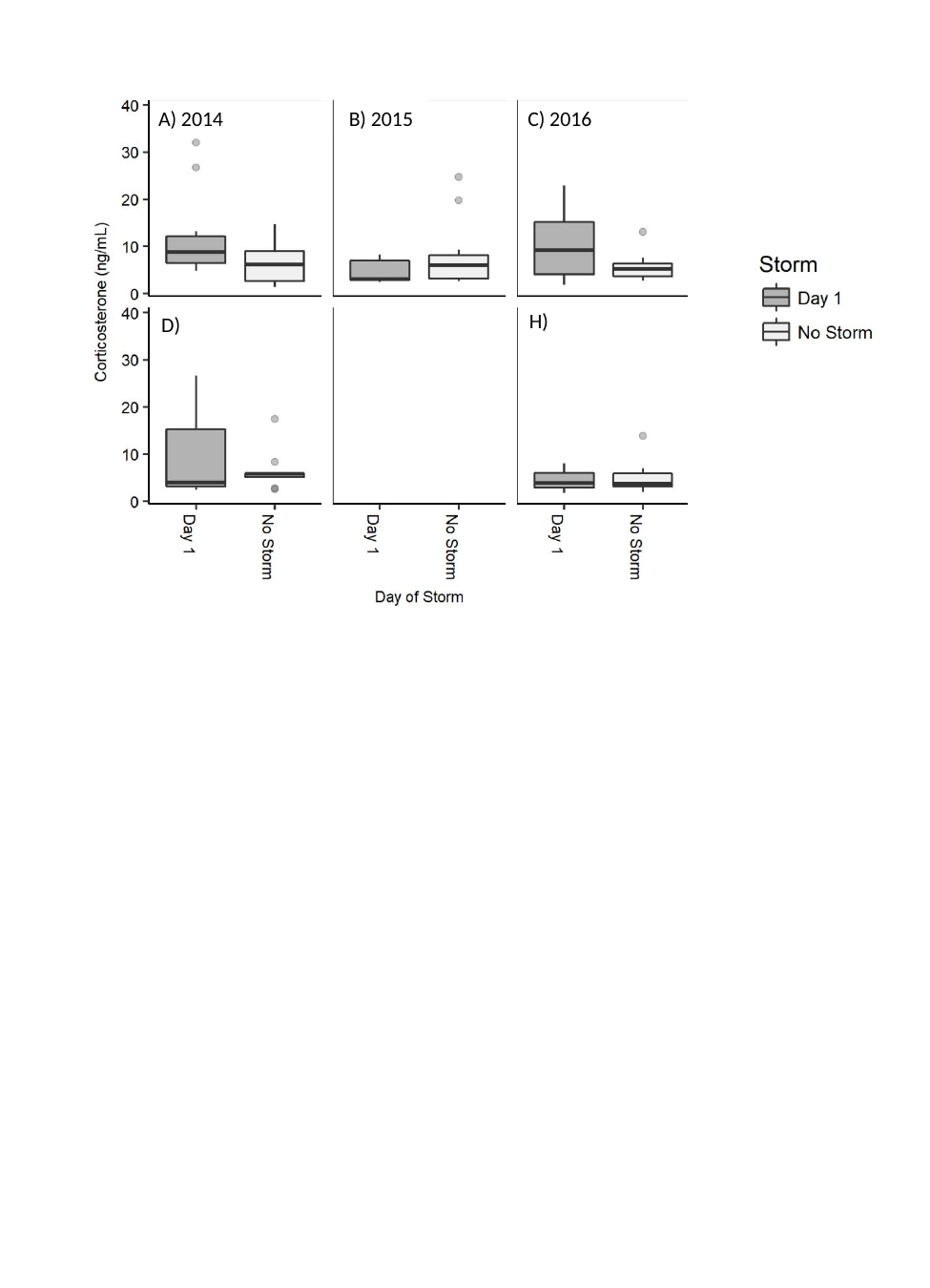

A) 2014
B) 2015
C) 2016
H)
D)

Supplement: Supplementary data 2 [file mmc2.pptx]
